# Illustrations of the Comparative Anatomy of the Nervous System

**Published:** 1837-04

**Authors:** 


Art. XIII.—*Illustrations of the Comparative Anatomy of the Nervous System.* By Joseph Swan. Part II.—*London,* 1836. 4to. pp. 24.

The second part of this valuable work is no less creditable than the first to the author and to his country, and fully supports the very high character which Mr. Swan holds as an anatomist of the Nervous System. In our former notice of this work, we felt it our duty to make a few strictures upon that portion of it which illustrates the anatomy of invertebrated animals, and to question the correctness of some of Mr. Swan's opinions respecting the analogies of the structures he was then describing, as not being in accordance with the received opinions and established facts in comparative anatomy. On the present occasion, we are happy to find less cause for criticism.

The Part which is now before us commences with a splendidly executed plate of the brain of the cod, (*Gadus morrhua,*) which forms a continuation of the anatomy of fishes from the preceding Number. Some very interesting distributions and connexions of nerves are shown in this dissection; as the connexion between the vagus and posterior branch of the fifth nerve by a branch from the former; the distribution of the third nerve, which gives branches to all the muscles of the eye, except the superior oblique and abductor, and sends ciliary branches to the interior of the organ; and the distribution of branches from the anterior branch of the fifth to the interior of the nasal orifices. These are interesting communications of nerves, but the origins of the nerves themselves are still more so. Thus, with the exception of the olfactory, optic, and sympathetic nerves, all the nerves of special and organic functions,—as the fourth, fifth, auditory, and vagus,—originate from the track of the restiform body; while the true motor nerves, the third and sixth, always arise from that of the pyramidal body, precisely analogous to similar nerves in the human body. In the ninth plate, the second of this part, there is a beautiful delineation of the sympathetic nerve and of the gastric branch of the vagus in the skate, (*Raia batis.*) The distinguishing anatomical characters of these two nerves are well shown: the vagus nerve is entirely without ganglia, and distributed over the sides of the stomach; while the sympathetic, which is given to the whole interior of the body, is largely supplied with them. This exposition of the sympathetic nerve is the more interesting from the circumstance that, until within these few years, the existence of a sympathetic nerve in fishes was doubted; and from the remark of Professor Müller, that ganglia are absent in the sympathetic system of most fishes; that they are not essential to constitute the sympathetic system. The description which Mr. Swan gives of the sympathetic nerves of the skate is so distinct, that we shall quote it in his own words:

"On each side of the superior part of the abdomen, at a short distance from the spine, the sympathetic nerve forms an unequal oblong ganglion, of a reddish-ash colour; it gives off both very large and very small nerves, having this appearance, to pass on the mesentery, communicate with branches of the par vagum, and accompany the mesenteric arteries to the viscera. Some filaments are distributed about the testicles, and others pass towards the aorta; but these are too soft to be satisfactorily traced to their precise terminations.

"The large ganglion communicates by a semi-transparent tissue with a small one; this with the next, and so on to some distance down the spine; it communicates by the same tissue with the par vagum, and the large nerves collected from the spinal cord, which resemble the axillary plexus of the higher classes. It may be a question whether this semitransparent tissue be nervous; but, as it connects small ganglia with each other and with the large one, but little doubt can be entertained respecting its true character, notwithstanding the nerves proceeding from the large ganglion to the viscera have a different appearance.

"The sympathetic nerve of this creature must be formed almost entirely for the abdominal viscera, as similar ganglia have not yet been observed in any other part of the body. There is, indeed, a ganglion attached to a branch of the fifth, and placed underneath the skin of the lower jaw, near each angle of the mouth; but it is almost transparent, and corresponds more with that of the gustatory nerve in some of the mammalia." (P. 33.)

From these facts, we think, there can be but little doubt that the true sympathetic system of fishes is perhaps as extensive as in the higher animals; the delicacy of the structure only rendering its finer ramifications of difficult detection. In the tenth plate, the brain of the skate, the pathetic nerve, is seen to arise "just behind the posterior point of the optic lobes;" and the fifth has two origins, "one from the restiform body, the other from a pedicle or process of the lateral lobe of the cerebellum."

The remaining plates illustrate the anatomy of the turtle, but, in our opinion, are not quite equal to those which illustrate the anatomy of the skate, particularly the splendid one which represents the whole fish.

We think Mr. Swan labours under a slight misconception with regard to the principles upon which the nervous system is developed. It is not to be imagined, as he appears to believe, that comparative anatomists are endeavouring to inculcate the absurd doctrine, that the human brain is really at one time exactly like that of a fish, of a reptile, or of a bird; but only that, during certain periods of the fœtal state, it is in a somewhat similar condition, or state of organization, to the brain of these animals; and not that it absolutely passes through their permanent forms. But these are trifling blemishes in a work of such excellence; and we beg again to recommend it to our readers in strong terms. The present Part, indeed, while equally valuable, will be found much more useful to the student of the anatomy of the nervous system than its predecessor.

